# Design and validation of a modular smart headband with embroidered electrodes for comfortable EEG monitoring

**DOI:** 10.1371/journal.pone.0347189

**Published:** 2026-09-09

**Authors:** Komal Komal, Frances Cleary, Ram Prasadh Narayanan, John S. G. Wells, Marco Buiatti, Louise Bennett

**Affiliations:** 1 School of Health Sciences, Southeast Technological University, Waterford, Ireland; 2 Walton Institute, Southeast Technological University, Waterford, Ireland; 3 Center for Mind/Brain Sciences, University of Trento, Rovereto, Italy; PLOS, UNITED KINGDOM OF GREAT BRITAIN AND NORTHERN IRELAND

## Abstract

**Background:**

Wearable electroencephalography (EEG) systems enable neural monitoring beyond controlled laboratory environments. However, conventional EEG devices are limited by bulky hardware, lengthy setup procedures, electrode-related discomfort or irritation, and high costs, restricting their suitability for long-term real-world use.

**Objective:**

This study presents the design and validation of a modular smart EEG headband integrated with soft e-textile electrodes for comfortable, reliable out-of-lab EEG acquisition suitable for long-term neural monitoring**.**

**Methods:**

This research developed six adjustable, replaceable embroidered electrodes (textrodes) incorporating a foam layer to improve scalp contact. These textrodes were embedded in a neoprene headband and positioned over the frontal and temporal regions just below the hairline. The performance of the smart headband in detecting spectral and event-related potential (ERP) effects in three standard tasks, such as eyes open/closed, auditory oddball, and visual oddball, on ten healthy participants, was validated and compared with a commercial system endowed with sponge-based wet electrodes. Comfort and usability were assessed via a standardized questionnaire.

**Results:**

The smart headband reliably captured alpha-band modulation during the eyes open/closed task (*p* = 0.01) and significant ERP effects in both the auditory and visual oddball tasks (*p* = 0.014 and *p* = 0.013, respectively), demonstrating performance comparable to the commercial system. Questionnaire results indicated superior comfort and the elimination of skin irritation for the smart headband.

**Conclusion:**

The smart headband demonstrates detectability of spectral/ERP functional responses comparable to a commercial sponge-based EEG cap, while substantially improving user comfort.

**Significance:**

By combining soft embroidered electrodes with a wearable, modular form factor, this study advances wearable EEG technology toward comfortable, cost-effective applications, aligning with emerging needs in real-world neuromonitoring.

## I. Introduction

Electroencephalography (EEG) is a non-invasive technique for investigating brain function and pathology by recording the electrical activity of neural origin from the scalp [1]. Thanks to recent technological advances, wearable EEG devices present novel opportunities for product development and therapeutic solutions for pathological conditions [2–4]. Given the non-invasive, real-time monitoring of EEG signals through wearable technology, several studies utilize EEG data features and observable patterns to interpret brain signals, thereby enabling the representation of neural activity evoked by external stimuli [5–8]. However, current wearable EEG acquisition systems often involve numerous wires, multiple gel-based or metallic electrodes, and bulky amplifiers [9–11]. The process of electrode placement and scalp preparation is time-consuming, and prolonged use of such complex devices for extended monitoring has been reported as uncomfortable and impractical for the wearer [12]. Furthermore, unstable contact between the scalp and the electrodes increases impedance and affects signal quality [13]. To achieve a reliable signal, additional pressure is often required to improve electrode contact; however, applying this pressure can contribute to wearer discomfort [10].

Clinical EEG systems still rely on medical-grade Ag/AgCl electrodes to detect electrical activity from the scalp surface [14]. The application of these electrodes requires an electrolyte gel to ensure good contact with the scalp, reducing skin–electrode impedance and providing mechanical stability [15]. These electrodes necessitate time-consuming skin preparation, and the electrolyte gel tends to dry out over time, increasing susceptibility to interference, signal degradation, and a reduced signal-to-noise ratio (SNR) [16,17]. Additionally, prolonged use of gel-based electrodes can cause skin irritation, such as rashes and allergic reactions [18]. Recent advancements in dry metallic disc electrodes have addressed some limitations of gel-based electrodes [19,20]. Dry electrodes are easier to apply, eliminate the need for gel, and enhance overall system usability [4,18]. Nonetheless, these dry electrodes typically exhibit higher electrode–scalp impedance than gel-based counterparts [21]. Moreover, their rigid structure is often uncomfortable for the wearer, and it can result in an unstable contact with the scalp, increasing the risk of motion artifacts and noise, which may degrade signal quality [4]. The development of a sponge-based commercial EEG cap, which uses soft sponges soaked in a saline solution, has further improved usability [22]. These commercial EEG caps enable faster preparation compared to traditional headsets while delivering equivalent or superior signal quality. They also offer improved comfort and make it easier to adjust electrode impedance for accurate EEG measurement. However, they are expensive, and still depend on a wired amplifier, which limits individual mobility and can be inconvenient due to managing and carrying cable connections [22].

In recent years, various textile-based electrodes have been developed using soft materials such as polymers, graphene, conductive inks, mask deposition, laser printing, smart conductive fabrics, and embroidery techniques [23–29]. The key advantages of textile-based electrodes are to record electrical activity without requiring contact materials (gel or saline solution), are very comfortable to wear, and are generally inexpensive. There remains a need for reliable textile-based EEG technologies that enable long-term monitoring while prioritizing wearer comfort and improving usability [13,30]. Although initial attempts have been made to compare their signal quality with medical-grade electrodes [31–34], most studies lack comprehensive validation of textile-based electrodes for real-world EEG applications, such as using them to assess cognitive brain function [28,35,36].

This paper directly addresses the limitations and gaps by designing and developing an innovative, low-cost, modular smart headband with six integrated embroidered electrodes (textrodes) for EEG recording and by comparing real-time data acquisition with a gold-standard wet-electrode system. The proposed smart headband improves wearer comfort, and its modular design can be adjusted to fit different head sizes, reducing the need to purchase multiple commercial EEG caps. It is easy to wear and eliminates the bulkiness caused by the multiple wires and external amplifier used in traditional EEG caps. The integration of an embedded Enobio 20 controller [37], makes the system portable and enables wireless transmission of EEG data. In addition, no skin preparation is required for wearing smart headbands, reducing the skin preparation time and also eliminating the risk of skin irritation or allergic reactions. Overall, the smart headband improves usability, wearability, and comfort, making long-term monitoring of EEG measurements more practical for the wearers.

A second key objective of this study is to address the gap in the validation of textrode by evaluating the performance of the proposed smart headband in assessing cognitive brain function and wearer comfort. The performance of the smart headband was validated by: (1) testing its efficacy in obtaining reliable neural responses during eyes open/closed rest, and classical cognitive tasks (auditory and visual oddball) in comparison with a commercial sponge-based wet-electrode EEG cap; and (2) assessing its comfort, wearability, and usability relative to the commercial EEG cap.

## II. Design & implementation

This section describes the fabrication of textrodes, the design and creation of the smart headband, and the EEG measurement setup, including the hardware and software implementation.

### a. Fabrication of embroidered electrode (textrodes)

A textile-based 3D textrode was designed and fabricated using the ZSK technical embroidery machine integrated with EPC Win software [38]. The design image created using the EPC Win software defined multiple parameters, such as shape, stitch pattern, stitch length, and stitch distance. Multiple textrode designs with variations in structural parameters were fabricated and characterized through electrical impedance analysis and mechanical testing, including washing and strength testing for failure analysis, to identify the optimal architectural design for garment integration [39]. The selection of the optimal textrode design was informed by electrode-skin impedance characterisation conducted in a prior dedicated study [39]. Electrode-skin impedance was measured by placing all textrode samples on the forearm and recording impedance across a frequency range of 10.1 Hz to 1 kHz. Among the fabricated embroidered textrode samples, the C2 cross-stitch pattern demonstrated the lowest impedance (50 kΩ/cm^2^ compared to 10 kΩ/cm^2^ for medical-grade Ag/AgCl electrodes) and the most suitable electrical contact performance [39]. The C2 design retained the same structural parameters as the prior study: a diameter of 20 mm, stitch length of 2.2 mm, and stitch distance of 2.5 mm. A key modification was introduced with the inclusion of a foam layer between the baseline fabric and the multi-layered embroidery stitches to elevate the electrode surface and improve skin contact. This modified three-dimensional (3D) design was used in the current study.

**Fig 1:**
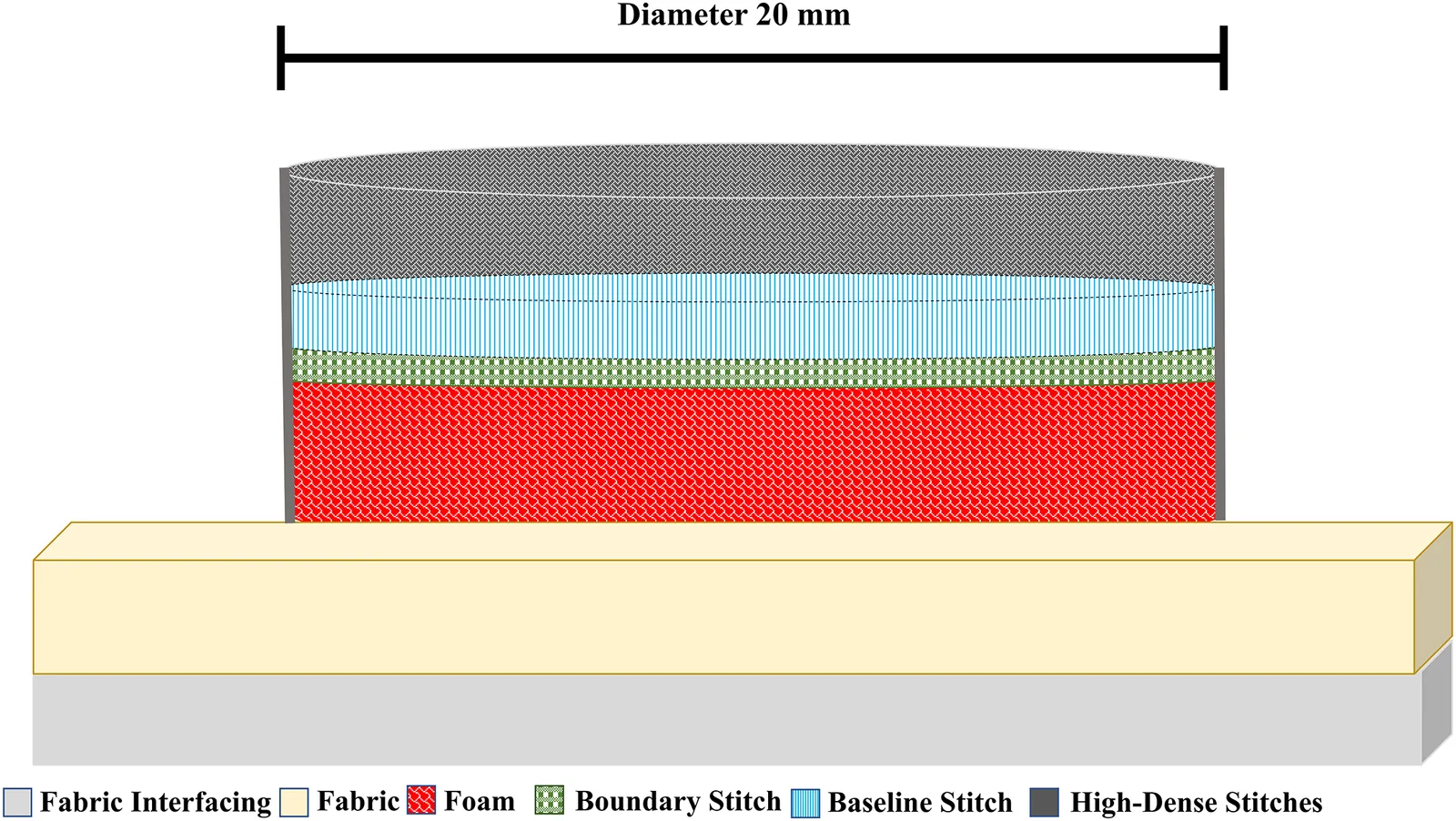
Multi-layered 3D textrode.

The software design of the 3D textrode consists of three stitch layers: (1) a boundary layer to define the shape of the electrodes; (2) a stabilizing middle layer with non-compact baseline stitches; and (3) a final stitch layer of conductive electrode stitches for biopotential measurements on the scalp. The fabrication of a multi-layered 3D textrode is presented in Fig 1.

Furthermore, the fabrication process of the 3D textrode may also comprise three distinct layers: (1) a calico 100% cotton baseline fabric along with an interfacing layer; (2) a 2 mm thick foam layer (commonly used for crafting due to its ease of cutting and adapting to customized shapes of the electrode [40], to raise the 3D effect to enhance scalp-electrode contact for accurate EEG measurements); and (3) the conductive stitch sub-layers for the measurement of biopotentials. The Madeira HC-12 conductive thread was employed for the fabrication of textrodes, whose resistivity is less than 100 ohms per meter [41].

### b. Creation of a smart headband modular design

The smart headband was constructed using a neoprene sports band (length 60 cm, width 8.5 cm at the edges and 5 cm at the center, fabric thickness 3 mm, and weight approximately 20 g), suitable for most adult head sizes [42,43]. Neoprene was selected due to its excellent fixation properties and its hair-holding ability, ensuring stability during physical activities. Furthermore, neoprene features an inner lining to enhance comfort and a velcro fastener at the end, allowing easy adjustment and removal.

A total of six 3D textrodes were integrated into the smart headband, positioned according to the 10–20 international electrode placement system at locations TP7, T7, FP1, FP2, T8, and TP8 [12,44,45], chosen to capture alpha activity and temporal responses to the auditory and visual stimuli tasks [12,13,44].

**Fig 2:**
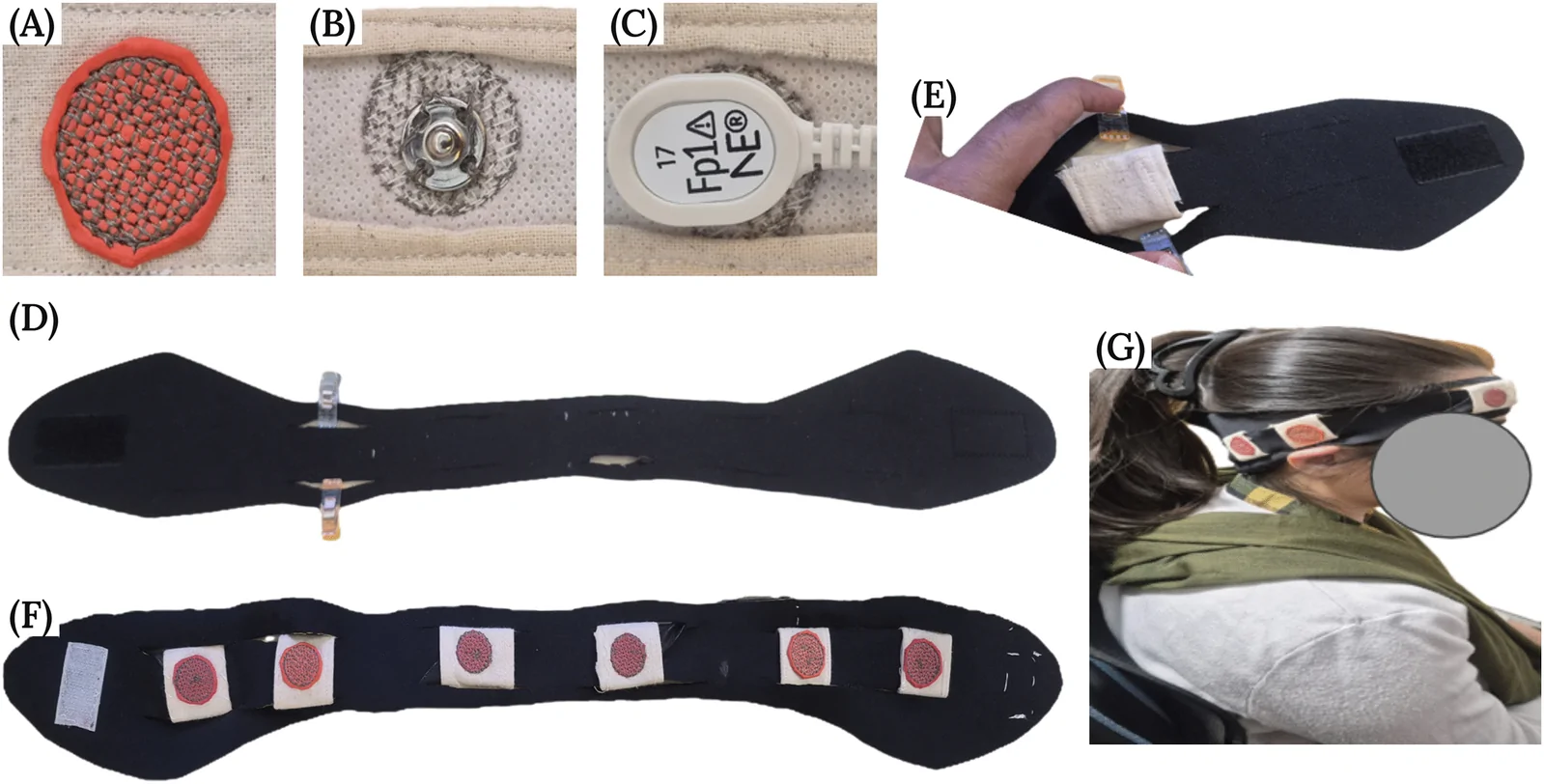
Smart headband embedded with textrodes. (A) Textrode module (front view), (B) Inner view (snap fastener for signal measurement), (C) Integration of the textrode with hardware wires, (D) Inclusion of twelve slits (six pairs) within the neoprene headband for electrode placement; (E) Fixation of the textrode module within the slit, (F) Full headband integrated with textrode, and (G) Smart headband worn on the participant.

Fig 2 illustrates the step-by-step creation of the smart headband design. Within the modular headband architecture design, the basic module consists of a 3D textrode fabricated on a 100% calico cotton baseline fabric (Fig 2A). Each fabric-integrated textrode module was cut to 100 × 50 mm, and its edges were hemmed with white non-conductive nylon thread. The textrode measured 20 mm in diameter, with an additional 5 mm of surrounding calico fabric (in total 25 mm). The center of the textrode was sewn with a 13 mm conductive snap fastener with HC-12 conductive Madeira thread on the backside of the textrode, enabling the connection to external wires (Fig 2B & 2C). The conductive side of the textrode contacted the skin for EEG signal acquisition, while the snap fastened on the back side allowed the direct connection to the Neuroelectrics system controller for EEG measurements.

Six such prepared modules were housed in a smart headband to accommodate lateral adjustment for different head sizes while maintaining a functional interface for EEG measurement (Fig 2D–2F). The smart headband EEG was worn by one of the authors (see Fig 2G) for illustrative purposes only (this image does not depict a study participant). This modular design enables adaptability for individuals with different head sizes and facilitates easy electrode adjustment and replacement. The neoprene headband incorporates twelve horizontal slits (six pairs) of 40 mm-long slits along its 60 cm length, to accommodate textrode placement. The slits are positioned 1 cm from the top and bottom, respectively, leaving 3 cm vertical central space to house each textrode module (20 mm diameter of textrode surrounded by approximately 5 mm calico fabric). Each of the 25 mm wide textrode modules was housed within the slit and was secured with a velcro strip at its ends on the inner side of the headband. This configuration allows approximately 15 mm lateral adjustment for head-size adaptation and optimal 10–20 electrode positioning.

In addition, the velcro strip arrangement allows individual modules to be removed for washing or replacement in case of fraying or damage/degradation, without needing to replace the entire smart headband. This modular architecture design of the smart headband ensures comfort and adjustability, while maintaining stable electrode-skin contact. Overall, the slit-based smart headband design provides a low-cost alternative to traditional reusable gel-based electrodes and enhanced flexibility to accommodate different head sizes for long-term EEG measurements.

### c. EEG measurement setup

**Fig 3:**
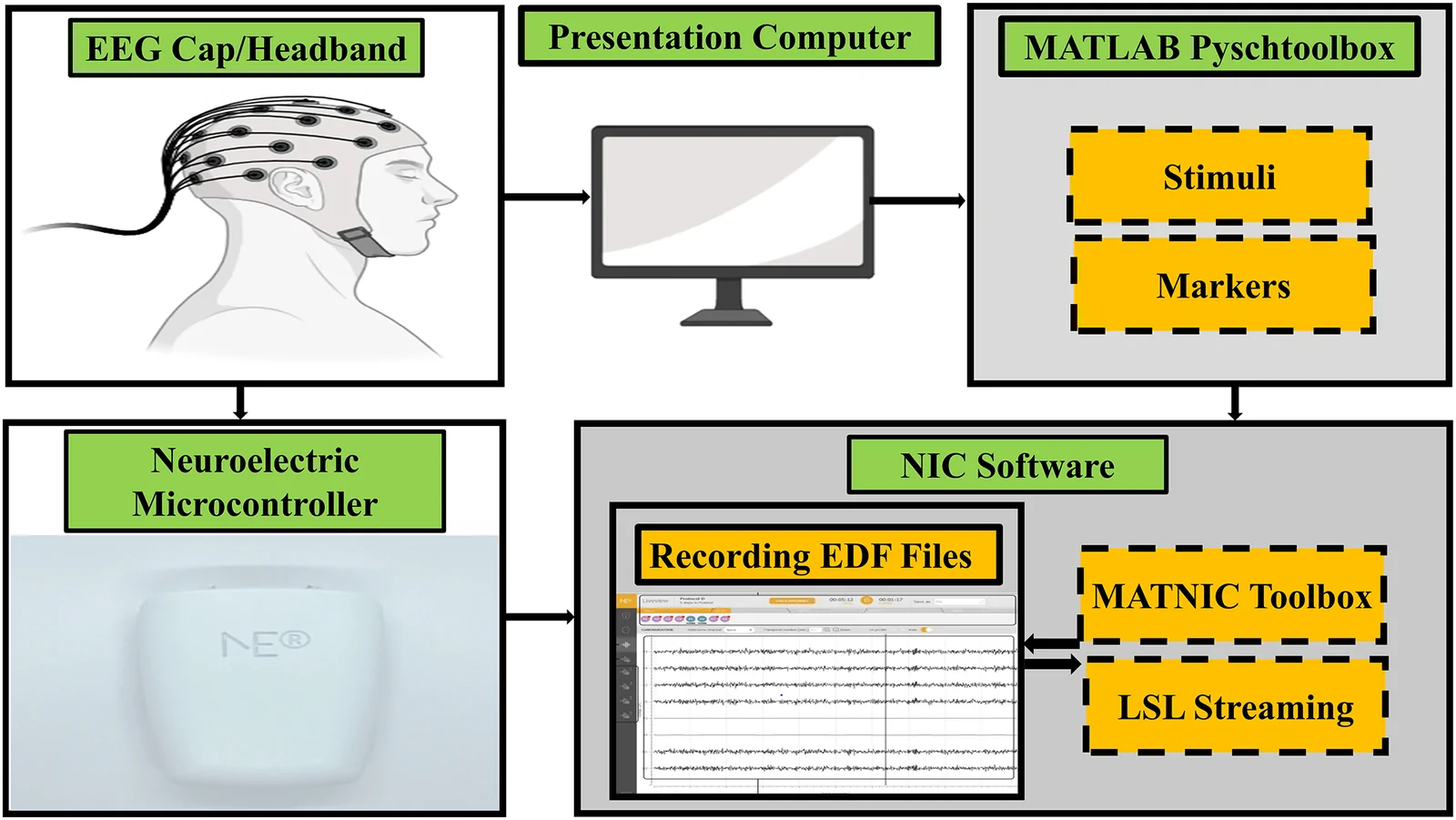
Workflow of EEG signal acquisition using the smart headband with textrodes, Enobio controller, and NIC software.

The smart headband EEG measurement setup involved various steps summarizing the hardware and software pipeline, from the electrode or textrode placement to the EEG recording data, as illustrated in Fig 3.

During the experimental session, electrical signals were detected on the participant’s scalp using the textrode-integrated smart headband. Textrodes were placed on the temporal sites in the forehead (TP7, T7, T8, TP8) and frontal (FP1, FP2), where little to no hair is present (hair increases contact impedance, which can degrade signal quality) [46], and no skin preparation was conducted, thereby reducing setup time and improving usability. The textrodes were connected to the Enobio 20 microcontroller via snap fasteners [47,48]. This microcontroller was secured to the participant’s headband with a velcro strip at the central back of the participant’s head for EEG measurements and wirelessly transmitted to the Neuroelectrics® Instrument Controller (NIC) software for recording the EEG data. The lightweight controller (89 mm × 61.21 mm × 23.8 mm and ~58 g) ensured that it caused no discomfort to participants during EEG experimentation [37].

Once the textrodes were correctly positioned, the functionality of the smart headband was verified by visualizing an EEG signal from the customized NIC software (through the Quality Index matrix, described below on the software side setup). Participants were seated in a dimly lit room on a comfortable chair, positioned 60 cm from a computer screen. They were instructed to watch the screen for the presentation and to follow the instructions related to the EEG experimental tasks. Details of the behavioral tasks are provided in Section III. b.

Fitting the smart headband, including the positioning of the modular embedded textrodes, took approximately 5 minutes, and performing the behavioral experimental tasks for EEG recording took 10–15 minutes; in total, the experiment took approximately 15–20 minutes.

In relation to the software side, stimuli and instructions related to behavioral tasks were presented on a display screen configured using Psychtoolbox 3.0.12 in MATLAB (The MathWorks Inc., Natick, Massachusetts, USA). An additional MATNIC toolbox was employed in the smart headband EEG recording to link the NIC software of the Enobio microcontroller with MATLAB’s Psychtoolbox through the Lab Streaming Layer (LSL) [49]. This MATNIC toolbox synchronizes the LSL stimulus presentation with event markers associated with the presentation stimuli and transmits these markers to the EEG recording within the NIC software. The raw EEG data were then recorded via the Enobio microcontroller and wirelessly transmitted to customized NIC software (see Fig 3).

Smart headband EEG was recorded using an 20-channel Enobio 20 wireless system (Neuroelectrics, Barcelona, Spain) controlled by NIC2 software. Signals were digitized at 500 Hz with 24-bit resolution (effective amplitude resolution 0.05 µV/bit) over a DC-coupled hardware bandwidth of 0–125 Hz. No hardware notch filter is present; the NIC2 software line-noise filter was set to 50 Hz. Data were written by NIC2 in easy ASCII format (channel values in nanovolts) and converted to EDF+ (channel values in microvolts, with NIC2’s high-pass filter applied during export); the unit conversion factor between the two formats is 1000.

Recording proceeded only when a green Enobio 20 NIC2 Quality Index indicator (QI < 0.5) was confirmed across all channels, as described in the Enobio manufacturer’s documentation. NIC2 QI provides signal contact quality assessment rather than a direct numerical impedance value. However, the latest version of Enobio manufacturer’s documentation specifies that for dry electrodes, a green QI indicator corresponds to impedance below 150 kΩ [50], which is fully consistent with the typical impedance values of the dry electrodes [51].

The same experimental setup was used to record the data from a commercial EEG system (Brain Products, Munich, Germany) [52]. This commercial EEG system consisted of 64 sponge-based electrodes, which were soaked in a saline physiological solution before scalp contact, requiring approximately 10–15 minutes for scalp preparation, electrode placement, and impedance adjustment. Once preparation was complete, we presented participants with the same experimental protocols used in the first phase through Psychtoolbox 3.0.12 in MATLAB. The total duration of the experiment for each participant using the commercial EEG system was approximately 30 minutes, excluding break times.

A commercial EEG cap was recorded using a BrainAmp amplifier (Brain Products GmbH, Gilching, Germany) with the passive Ag/AgCl sponge-based R-Net electrode system, controlled by BrainVision Recorder. Signals were digitized at 500 Hz with 16-bit, 0.1 µV/bit, ± 3.28 mV range. The hardware bandwidth was 0.016 Hz – 250 Hz; no hardware notch filter was used (the amplifier is battery-powered, eliminating mains-coupled noise). During all the recording sessions with the commercial sponge-based EEG cap, electrode impedances were kept below 50 kΩ, consistent with standard wet-electrode EEG practice.

At the end of the experiment, the commercial cap was removed, and participants were offered the opportunity to wash their heads in a bathroom adjacent to the experimental room. The sponge-based EEG electrode system was rigorously sterilized after each participant’s session.

## III. Validation of the smart headband performance

Following ethical approval and participant recruitment, the performance of the smart headband was validated by: (1) evaluating its ability to measure neural responses during behavioral tasks; and (2) assessing its comfort through the administration of a survey examining EEG measurement for the wearer’s comfort.

### a. Ethical approval and recruitment of participants

This observational signal validation study was conducted and reported in accordance with the STROBE guidelines for observational research [53]. Signal comparability between the smart headband and the reference commercial EEG cap was assessed by evaluating Alpha wave, ERP peak amplitude, and latency values across conditions using established comparison methods. The study validating the smart headband performance was conducted at the Center for Mind/Brain Sciences (CIMeC), University of Trento, Italy, and involved both undergraduate and postgraduate students. The recruitment period for this study ran from 25/05/2024–30/06/2025. Ethical approval was obtained from the Southeast Technological University (SETU), Waterford, Ireland **(**Ref: SETUREC23/24/044**),** and the University of Trento, Italy. Participants willing to participate received an information leaflet detailing the study procedures.

Ten participants (six females, four males; mean age 32.2 ± 10 years) provided written informed consent in Italian language from May 2024 to June 2025. Participants were informed of their right to withdraw from the study at any time without consequence. Personally identifiable information was accessible only during data collection to allow withdrawal if requested. There were no deviations from the approved study protocol during the conduct of the trial. All procedures were followed in accordance with the approved ethical guidelines and study protocol. All EEG recordings and comfort assessment questionnaires were anonymised prior to preprocessing and analysis. This study followed ethical, cultural, and scientific considerations specific to inclusivity in global research throughout the research process.

### b. Experimental protocol

**Fig 4:**
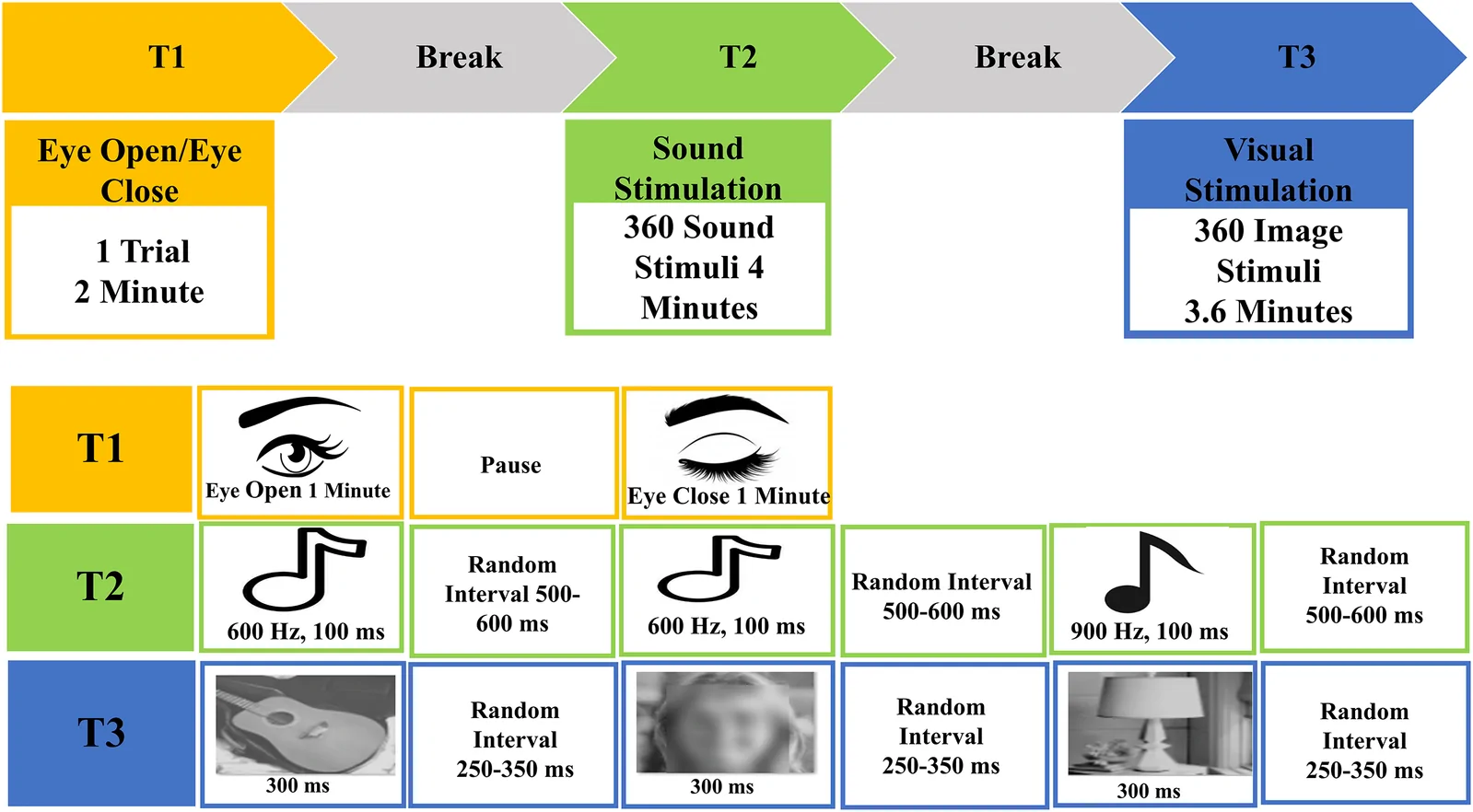
Tasks (T1-T3) and their sequence within the experiment.

Participants wore smart headbands connected to the EEG acquisition system via the Enobio 20 controller and NIC software and were asked to sit comfortably while the setup was prepared. Then the experimental protocol commenced (Fig 4):

***Task protocol 1 (eyes open/closed):*** Participants were instructed to sit in a chair in a relaxed resting state with their eyes open for one minute. Subsequently, participants were instructed to close their eyes for 1 minute.

***Task protocol 2 (auditory oddball):*** Participants listened to a sequence of tones consisting of a 600 Hz tone (80% of the trial time) and a 900 Hz Sinewave deviant stimulus (20% of the trial time), each presented for 100 ms with an inter-stimulus interval (ISI) of 500–600 ms. A total of 360 trials were presented in a randomized order, divided into four blocks of 90 trials each. Breaks were provided between blocks to ensure the participant’s comfort, and the next block started when the participant was ready. During each block, to maintain focus and attention on the experiments, participants were asked to count the number of deviant stimuli they heard. This task lasted approximately 3–4 minutes, excluding breaks.

***Task protocol 3 (visual oddball):*** Participants were shown a series of images (the same used in the study [54] to assess face-sensitive EEG responses). A total of 360 images (trials) were presented and divided into four blocks of 90 images each. Two types of stimuli were used: standard stimuli (objects) and deviant stimuli (faces) [55]. The image sequence was block-randomized, with each image displayed for 300 milliseconds and with an ISI of 250–350 ms. The total duration of this task was approximately 3–4 minutes, excluding breaks. To maintain attention, participants were instructed to count the number of deviant (face) stimuli presented in each block. As in the auditory task, breaks were provided between blocks to maintain participant comfort.

### c. EEG data analysis

Raw EEG signals recorded during the experimental tasks with both the smart headband and the commercial EEG system were then processed using the same pipeline of data analysis.

#### i. Pre-processing pipeline.

The raw EEG data were imported into the EEGLAB software [56] and bandpass filtered between 0.3 and 30 Hz with the default EEGLAB filter to remove DC and high-frequency noise [56,57]. Of the eleven participants (n = 11) who underwent the EEG recordings, one was excluded prior to analysis due to excessive movement artefacts that rendered the EEG recording data unusable, resulting in a final sample size of ten (n = 10). All remaining participants completed the experimental protocol with usable EEG datasets for both the smart headband and the commercial EEG cap. The filtered EEG data were visually inspected, and intervals containing nonstereotyped paroxysmal artifacts were discarded. The proportion of rejected data did not differ systematically between the smart headband and commercial cap recording systems. Bad channels were identified with the LOF algorithm [57]. For the smart headband EEG, zero to a maximum of one channel was discarded, while for the commercial cap, on average 2–3 channels per participant were discarded.

To identify and remove stereotypical artifacts, the default EEGLAB Independent Component Analysis (ICA) decomposition was computed on the concatenation of all segments [56,58]. Blinks, eye movements, and other topographically localized artifacts were discarded by removing the corresponding independent components identified by visual inspection of their topography and spectro-temporal profile, supported by the classification provided by ICLABELS [58]. EEG signals in bad channels were interpolated with the EEG signals from neighboring channels (standard spherical interpolation method in EEGLAB). To properly compare the task-related EEG responses measured with the two systems, the commercial EEG data were re-referenced to the electrode channel (Fz), which was employed as a reference channel for the headband EEG data. The EEG data were then divided into three segments corresponding to each task (T1 – T3). For a fair comparison in the final analysis, this study selected only the electrodes from the commercial EEG cap that correspond to the locations of the textrodes in the smart headband*.*

#### ii. Data analysis.

The two EEG segments corresponding to the “eyes open” and “eyes closed” conditions (linked to Task 1) were selected for the analysis of alpha waves and further inspected to ensure that any remaining eye blinks or noisy intervals were identified and eliminated [44]. Following that, the power spectral density was analyzed to compare the eye-open and eye-closed conditions [18]. Tasks 2 and 3, associated with the auditory and visual oddball paradigm, were employed to assess cognitive performance by comparing the event-related potential (ERP) associated with the standard and deviant conditions [59,60].

In order to evaluate the ERP-related tasks (2 and 3), further post-processing steps were undertaken: (1) data were epoched time-locked to the onset of the stimuli (time range: [−0.1, 0.5] s); (2) baseline correction in the interval [−0.1, 0] s was applied; and (3) an automatic epoch rejection threshold of ± 100 µV was established to reject epochs containing residual excessive noise or movement artefacts. Epochs exceeding ± 100 µV on any channel were automatically rejected. Mean rejection rates for the auditory oddball paradigm were 18.1 ± 8.1% (smart headband) and 23.8 ± 13.0% (commercial cap). Mean rejection rates for the visual oddball paradigm were 22.1 ± 13.3% (smart headband) and 27.1 ± 9.2% (commercial cap).

#### iii. Statistical analysis.

First, group-level effects were assessed using cluster-based permutation testing. For Task 1, the statistical significance of the power spectrum difference between eyes-open and eyes-closed conditions was evaluated in the alpha range (8–12 Hz). For tasks 2 and 3, the oddball effects were evaluated through the statistical significance difference between the ERPs associated with the standard and deviant oddball conditions within the latency range of 0–500 ms, using the non-parametric cluster-based test [61] implemented in Fieldtrip [62]. This method allows statistical testing with no need for a priori selection of spatial ROIs because it controls for multiple comparisons by clustering neighboring channel pairs that exhibit statistically significant effects (test used at each channel point: dependent-samples t statistics, threshold: p = 0.05) and using a permutation test to evaluate the statistical significance difference at the cluster level (Montecarlo method, 1000 permutations for each test).

Second, within-device signal detection was quantified using Hedges’ g_z, a bias-corrected standardized effect size for paired within-participant designs [63,64]. Hedges’ g_z quantifies the magnitude of the difference between deviant and standard conditions in units of the within-participant, providing a scale-free index of how reliably each device detects the experimental effect independently of sample size [64]. For each electrode and each device separately, g_z was computed from the mean difference between deviant and standard amplitude across participants, normalized by its within-participant standard deviation, and corrected for small-sample positive bias using the factor J = (1 − 3/ (4n − 5)) [63]. Bootstrap 95% confidence intervals were derived from 5,000 resampling iterations with replacement, with n = 10 participants [65].

Third, inter-device agreement between the smart headband and the commercial EEG cap was assessed using Pearson correlation and ICC(A,1), a two-way mixed-effects intraclass correlation coefficient specifying absolute agreement and single-measure reliability [66,67]. Agreement was computed on cluster-constrained amplitude values rather than full-window per-electrode averages, yielding one value per participant per device prior to any agreement analysis. For the alpha paradigm, the eyes-closed minus eyes-open power was extracted within the 9.5–10.5 Hz range across all significant cluster electrodes. For ERP paradigms, the time point of maximum cluster-level statistics difference between deviant and standard stimuli was identified within the pre-specified analysis window across cluster electrodes. The mean amplitude was then extracted within a ± 15 ms window centered on that peak and averaged across the cluster electrodes. Pearson correlation and ICC(A,1) were then computed on these paired values, with bootstrap 95% confidence intervals derived from 5,000 resampling iterations [65].

### d. Survey on the comfort level of each EEG system

Following the EEG signal recording session, participants were invited to complete a questionnaire survey for assessing the comfort and wearability of the smart headband compared to the commercial EEG system. These closed-ended questionnaires included: firstly, to rank the overall comfort level of wearing the smart headband compared to the commercial EEG cap, with 1 indicating the lowest and 5 indicating the highest; secondly, to rate any discomfort or skin irritation experienced from wearing the smart headband textrodes on their forehead or when using the commercial EEG cap (scale from 1 (no irritation) to 5 (highest irritation)); thirdly, to describe the sensation they experienced on their forehead while wearing the smart headband with the microcontroller attached; and finally, if they felt stable and secure while wearing a smart headband on their foreheads.

## IV. Results

### a. Task 1 (eyes open/eyes closed)

**Fig 5:**
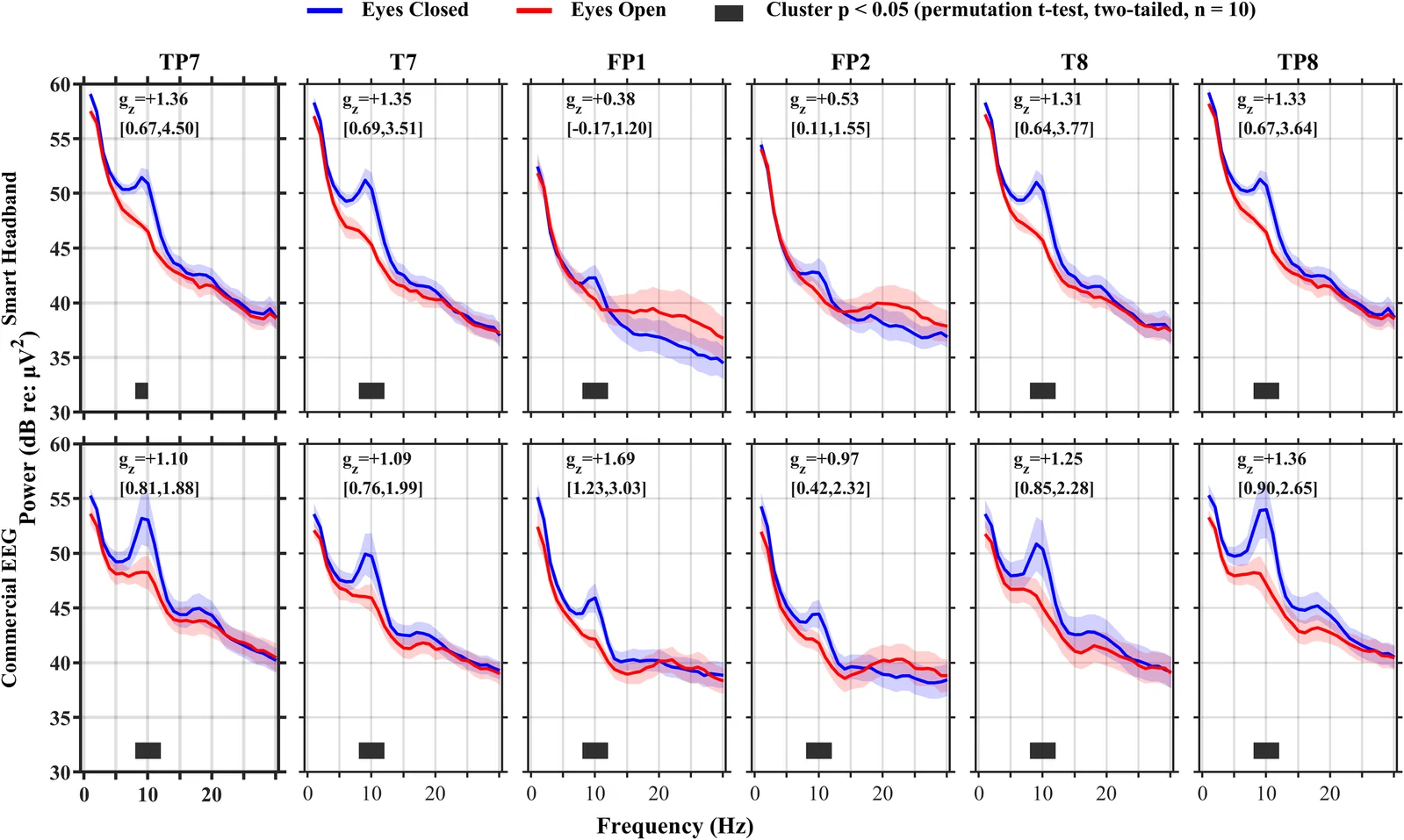
Task 1. **Both EEG systems detect the Alpha-band increase from eyes-open to eyes-closed.** (A) **Top Row:** EEG power spectrum from the smart headband, (B) **Bottom Row:** EEG power spectrum from the commercial headset. **X-axis**: Frequency in (Hz), **Y-axis**: Power Spectral Density in (μV^2^). The blue and red line graphs illustrate eye-closed and eye-open conditions, respectively. The thick-colored lines display the grand-averaged power spectrum, whereas the light-colored shaded area displays the standard error of the mean. For each electrode, the horizontal black bars indicate the latency intervals of the significant difference between eyes-opened and eyes-closed after the cluster-based statistical analysis. Hedges’ g_z values for each subplot indicate the within-device effect size, with a 95% bootstrap confidence interval (5,000 iterations), for the difference between deviant and standard amplitudes at each electrode.

Task 1 was designed to evaluate whether the smart headband could reliably detect the increase in the Alpha frequency band (8–12 Hz) from eyes-open to eyes-closed. The power spectrum of the EEG signal was computed separately for the smart headband and the commercial EEG cap during the eyes-open and eyes-closed sessions (Fig 5).

Group-averaged peak amplitudes of the Alpha waves were comparable between devices, ranging from 45–50 μV^2^ for the smart headband and 45–55 μV^2^ for the commercial cap, indicating similar signal acquisition performance. Clear alpha peaks were most prominent at TP7, T7, T8, and TP8 in both systems. Cluster-based statistical analysis of the difference in alpha-band power between the eyes-open and eyes-closed conditions (see Methods) identified a statistically significant positive cluster in both systems (smart headband: cluster *p_value* (hereafter indicated as *p*) = 0.015; commercial cap: *p* = 0.044), indicating reliable detection of the expected effect. The findings were consistent with previous literature [12,44,68], supporting alpha activity in eye closure for drowsiness detection-related neurofeedback applications [18].

Alpha band power agreement was assessed over the significant cluster window (9.5–10.5 Hz). Metrics were computed on cluster-averaged amplitude per participant, obtained by simultaneously averaging across all significant channels and the statistical cluster window. Effect sizes were quantified using Hedges’ g_z, Pearson r, and ICC(A,1) (two-way mixed-effects, absolute agreement, single measure), which were computed on the same cluster-average values, with bootstrap 95% confidence intervals (5,000 iterations) (Table 1).

**Table 1:** Inter-device agreement for alpha band (9.5–10.5 Hz) EC − EO power between the smart headband and commercial EEG cap (n = 10).

Metric	Effect size: Headband g_z [bootstrap 95% CI]	Effect size: Cap g_z [bootstrap 95% CI]	Inter-device agreement:Pearson r [bootstrap 95% CI]	Inter-device agreement:ICC(A,1) [bootstrap 95% CI]
**Alpha (9.5–10.5 Hz)**	**+0.987 [+0.50, + 2.13]**	**+1.081 [+0.79, + 1.79]**	**+0.782 [+0.29, + 0.96]**	**0.775 [+0.28, + 0.92]**

Both devices showed large increases in EC > EO alpha power across the significant cluster, confirming robust within-device detection of the alpha response (headband g_z = +0.987; commercial EEG cap gz = +1.081). Inter-device agreement was strong ICC(A,1) = 0.775 (r = +0.782), with confidence intervals entirely above zero, indicating consistent individual-level agreement between devices.

### b. Task 2 (auditory oddball)

**Fig 6:**
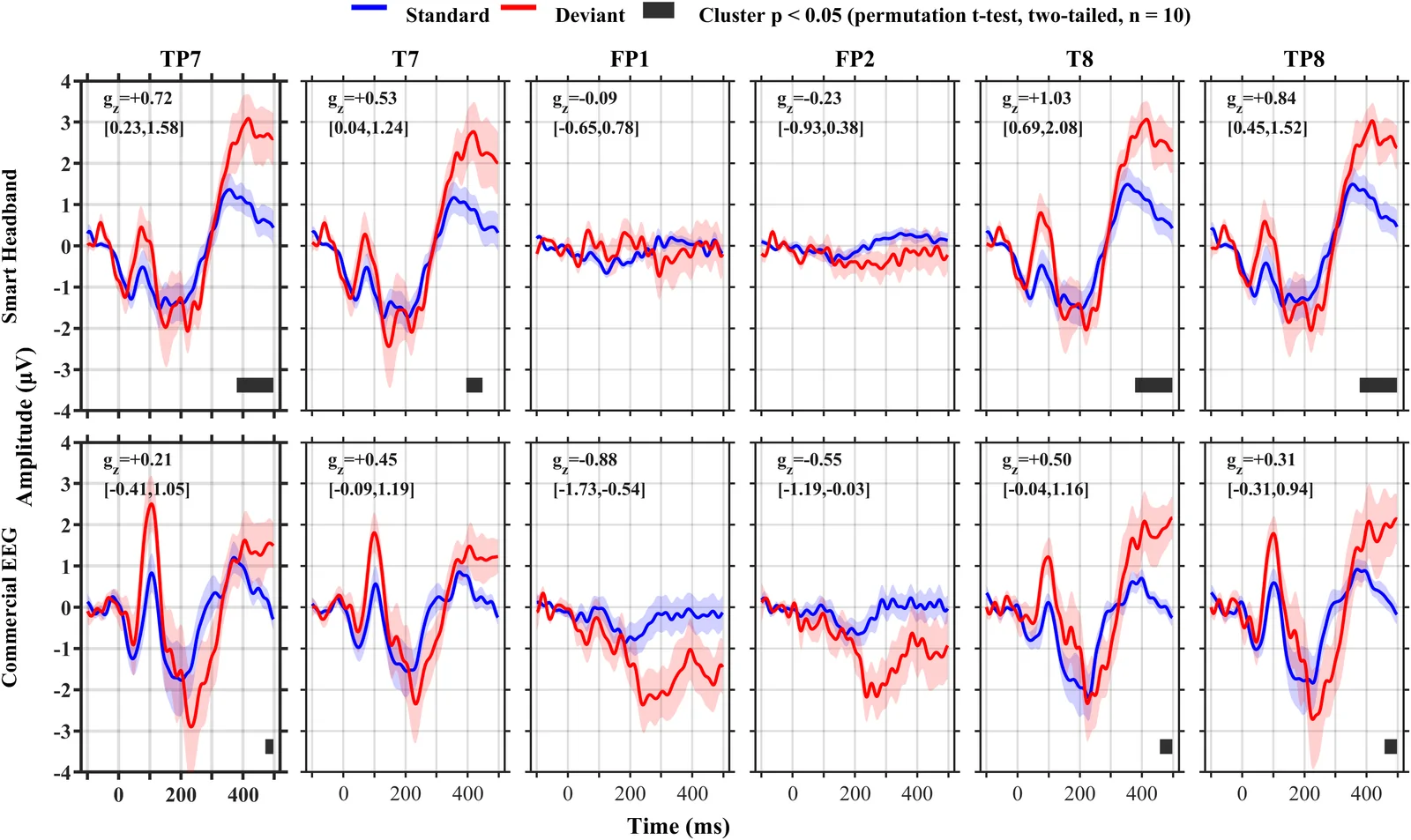
Task 2. **Both EEG systems detect the auditory oddball effect.** (A) **Top Row:** ERPs from the smart headband, (B) **Bottom Row:** ERPs from the commercial headset. **X-axis**: Time in (ms) relative to stimulus onset. **Y-axis**: ERP amplitude in (μV). The blue and red line graphs illustrate the ERPs from the standard and deviant stimuli responses, respectively. The thick-colored lines display the grand-average ERPs, while the light-colored shaded area represents the standard error of the mean. For each electrode, the horizontal black bars indicate the latency intervals of the significant difference between the standard and deviant stimuli after the cluster-based statistical analysis. Hedges’ g_z values for each subplot indicate the within-device effect size, with a 95% bootstrap confidence interval (5,000 iterations), for the difference between deviant and standard amplitudes at each electrode.

The smart headband reliably detected auditory ERPs at comparable latencies to the commercial EEG cap. To evaluate the auditory oddball effect, the P300 component was analyzed, as it is expected to be elicited by the deviant stimuli but not by the standard stimuli [69]. Fig 6 represents the auditory oddball ERP measured from the smart headband and commercial EEG cap.

The amplitude is similar across two systems, ranging from approximately −4 to +4 μV. While the ERPs from the smart headband are slightly noisier compared to the commercial EEG cap, an initial wave at around 100 ms and a clear P300 starting at 250 ms and peaking at 450–500 ms after stimulus onset are clearly visible in both systems. For the smart headband, the P300 difference between deviant and standard stimuli was statistically significant in a bilateral cluster including electrodes TP7, T7, T8, and TP8 (*p* = 0.014, latency: 370–500 ms). The commercial EEG cap also displayed an effect in the expected time window (*p* = 0.044, 458–498 ms), with significant electrodes at TP7, T8, and TP8.

Table 2 presents cluster-averaged effect sizes, Pearson r, and ICC for the auditory oddball condition. Effect sizes for the auditory oddball condition were comparable between devices, with headband g_z = +0.847 [+0.34, + 2.02] and commercial EEG cap g_z = +0.831 [+0.38, + 1.69], indicating similar within-device detection strength despite the wide confidence intervals expected at this pilot sample size (n = 10).

**Table 2:** Inter-device agreement for auditory oddball P300 (458-498 ms) between the smart headband and commercial EEG cap (n = 10).

Metric	Effect size: Headband g_z [bootstrap 95% CI]	Effect size: Cap g_z [bootstrap 95% CI]	Inter-device agreement:Pearson r [bootstrap 95% CI]	Inter-device agreement:ICC(A,1) [bootstrap 95% CI]
**Auditory P300 (458–498 ms)**	**+0.847 [+0.34, + 2.02]**	**+0.831 [+0.38, + 1.69]**	**+0.485 [+0.00, + 0.86]**	**0.508 [+0.00, + 0.76]**

Across the significant cluster electrodes within the stat-cluster window (458–498 ms), ICC(A,1) = 0.508 (r = +0.485). Despite individual-level concordance being more limited than alpha modulation, both systems detected the P300 at the group level, supporting the feasibility of the smart headband for capturing auditory oddball responses in this pilot cohort.

### c. Task 3 (visual oddball)

**Fig 7:**
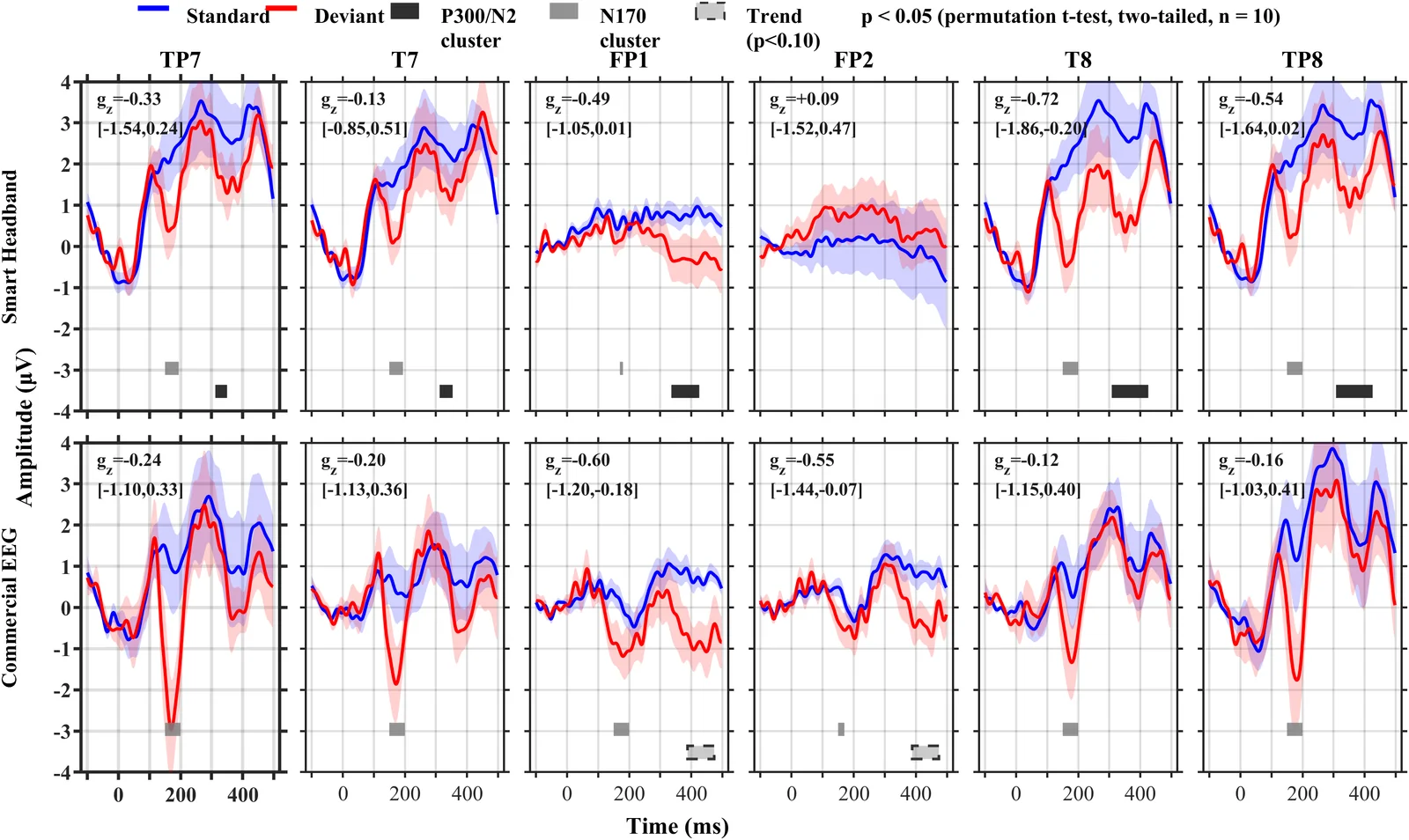
Task 3. **Both EEG systems detect the visual oddball effect.** (A) **Top Row:** ERPs from the smart headband, (B) **Bottom Row:** ERPs from the commercial headset. **X-axis**: Time in (ms) relative to stimulus onset. **Y-axis**: ERP amplitude in (μV). The blue and red line graphs illustrate the ERPs from the standard and deviant stimuli responses, respectively. The thick-colored lines display the grand-average ERPs, while the light-colored shaded area represents the standard error of the mean. For each electrode, the horizontal black bars indicate the latency intervals for significant differences between the standard and deviant stimuli after cluster-based statistical analysis. Hedges’ g_z values for each subplot indicate the within-device effect size, with a 95% bootstrap confidence interval (5,000 iterations), for the difference between deviant and standard amplitudes at each electrode.

In the visual oddball paradigm, deviant face stimuli elicited a negative N170 component followed by a P300 response. Fig 7 represents the visual oddball ERP measured with the smart headband and the commercial EEG cap. N170 was clearly observed over temporal electrodes, whereas the P300 appeared comparatively attenuated. This is expected, as the P300 typically exhibits maximal amplitude over centro-parietal midline regions (e.g., Cz), which were not covered by the smart headband because its lateral electrodes were placed outside the hairline. Consequently, P300 responses were reduced, while the N170 component was more prominent.

Given the temporal electrode coverage of the two systems, statistically significant N170 effects were detected at the TP7, T7, T8, and TP8 electrode cluster for deviant face stimuli in the smart headband (*p* = 0.013) and the commercial EEG cap (*p* < 0.001). A robust negative deflection emerged approximately 100 ms post-stimulus and peaked between 150 and 200 ms, corresponding to the canonical N170 component in both systems. The N170 is a face-sensitive ERP component typically maximal at occipito-temporal sites and originates from lateral temporal regions posterior to the ears. A weaker but significant effect was also found within the P300 latency for each system (smart headband: *p* = 0.039, latency 306–410 ms; commercial cap: *p* = 0.043, latency 388–474 ms). In addition, a non-significant trend cluster (light grey bar) was observed at frontal sites FP1 and FP2 in the commercial EEG cap (*p* = 0.057, 388–474 ms), suggesting a possible but inconclusive frontal response within the P300 window.

Overall, both EEG systems detected a significant early effect associated with the face-related N170 wave, while the standard late oddball effect was weaker due to the lateralized electrode configuration, which is sub-optimal for the spatial distribution of the P300. Table 3 presents cluster-averaged effect sizes, Pearson r, and ICC for the visual P300/N2 and N170 windows. The headband showed a larger effect (g_z = −0.764) than the cap (g_z = −0.221) in this window.

**Table 3:** Inter-device agreement for visual oddball N2/P300 (334–362 ms) and N170 (156–184 ms) between the smart headband and the commercial EEG cap (n = 10).

Metric	Effect size: Headband g_z [bootstrap 95% CI]	Effect size: Cap g_z [bootstrap 95% CI]	Inter-device agreement:Pearson r [bootstrap 95% CI]	Inter-device agreement:ICC(A,1) [bootstrap 95% CI]
**Visual P300/N2 (334–362 ms)**	**−0.764 [−2.26, −0.20]**	**−0.221 [−1.10, + 0.35]**	**+0.744 [−0.01, + 0.97]**	**0.662 [−0.01, + 0.86]**
**Visual N170 (156–184 ms)**	**−0.788 [−2.22, −0.20]**	**−1.593 [−3.13, −1.08]**	**−0.172 [−0.73, + 0.72]**	**−0.167 [−0.69, + 0.64]**

For the P300/N2 window, amplitudes were averaged across significant cluster electrodes within the stat-cluster window (348 ± 15 ms), ICC (A,1) = 0.662 (r = +0.744). For P300/N2, the smart headband showed a significant negative cluster consistent with an N2 response; neither device produced a positive P300 cluster, suggesting the visual oddball paradigm at these electrode locations primarily elicited a frontal N2 rather than a classic parietal P300.

For the N170 window, amplitudes were averaged across the significant cluster electrodes within the stat-cluster window (156–184 ms). ICC(A,1) = −0.167 (r = −0.172), despite strong group-level N170 detection in both devices. The absence of individual-level agreement despite reliable group effects is consistent with the known spatial sensitivity of the N170 component.

### d. Assessment of comfort

**Fig 8:**
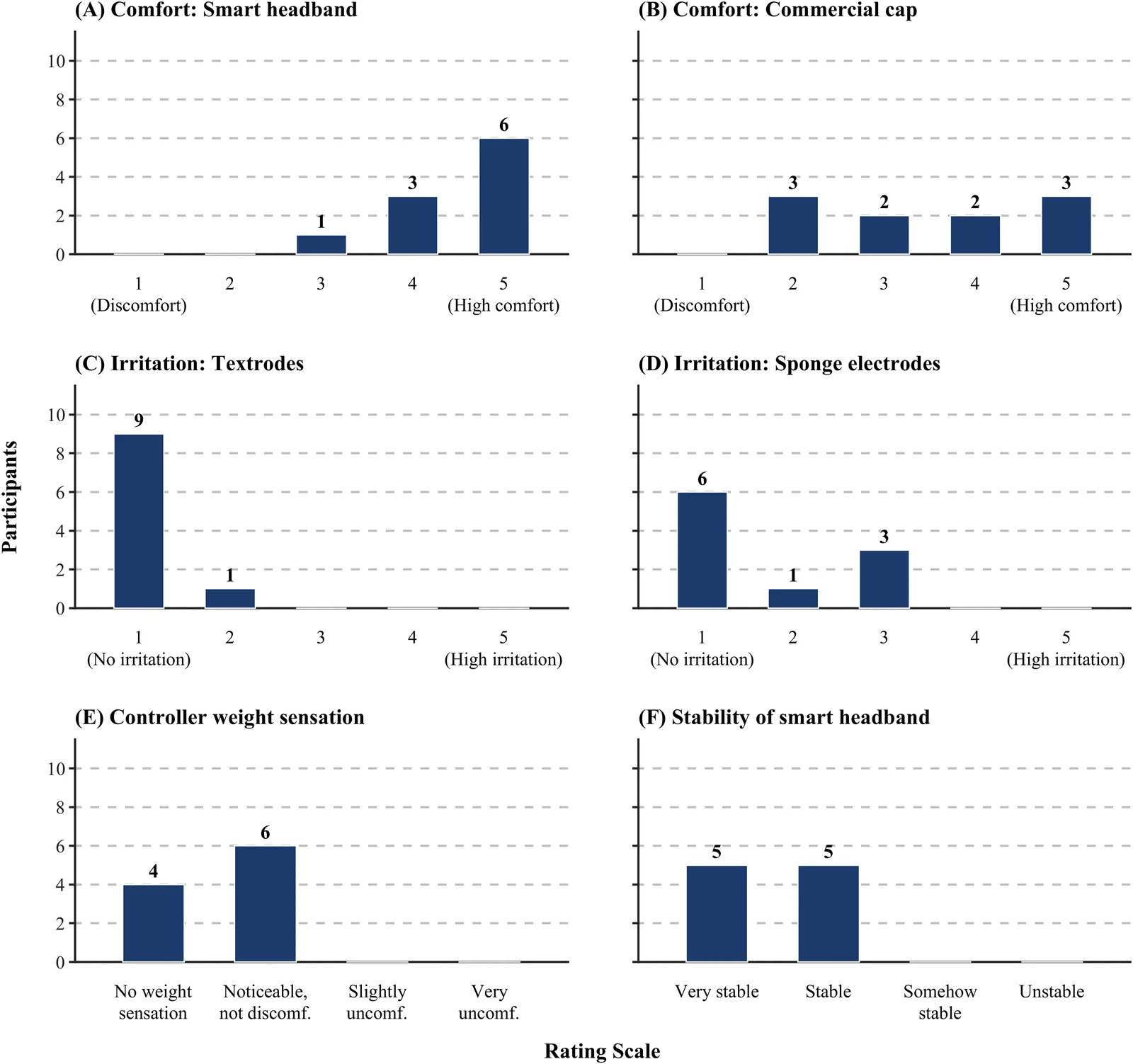
Survey results in relation to the comfort level of both EEG setup. (A) Comfort: Smart headband, (B) Comfort: Commercial cap, (C) Irritation: Textrodes, (D) Irritation: Sponge electrodes, (E) Sensation of controller weight of smart headband, and (F) Stability of smart headband.

The survey assessing the comfort level of the smart headband compared with the commercial EEG cap is presented in Fig 8. The participants were asked to rate their global comfort level regarding the EEG setup, which includes aspects such as wearing the EEG device, preparation time, carrying the controller at the back of the head, and overall wearability during the EEG recording session.

For evaluating the global comfort level of both systems, out of ten participants, six rated their comfort level as high (5), while three selected (4), indicating a high level of comfort. Only one participant rated their comfort level as 3, reflecting a medium level of comfort with the smart headband, and no one selected the lowest or minimum level of comfort (see Fig 8(A)). In contrast, the same question was posed regarding the commercial EEG cap presented in Fig 8(B). Three participants rated their comfort level as high (5), two selected (4), and two participants chose a medium level of comfort (3). However, three participants perceived the commercial EEG cap as having the lowest level of comfort by selecting (2). Fig 8(C) presents the findings related to the questions posed regarding skin irritation or discomfort related to electrode placement on the scalp. For the smart headband, nine participants reported no skin discomfort or skin irritation (max: scale level 1), while one reported minimal discomfort or skin irritation (scale level 2). However, the commercial EEG cap illustrated in Fig 8(D), in which six participants rated their experience as no skin irritation or discomfort (max: scale level 1), one participant reported minimal discomfort or skin irritation (scale level 2), while three participants indicated a moderate level of discomfort or skin irritation (medium: scale level 3).

In relation to controller weight sensation for the smart headband, responses consistently favored wearing the smart headband; four participants reported no weight sensation, while six reported noticeable weight but not discomfort when wearing it (see Fig 8(E)). Regarding a smart headband stability, five participants reported it as very stable, while five reported it as stable (see Fig 8(F)).

Survey results consistently favored the smart headband, with six out of the ten participants rating its comfort as ‘high’. The findings indicated no discomfort or irritation, with participants reporting positive feedback on secure wearability and stability. The only notable sensation reported was a mild perception of the microcontroller’s weight.

## V. Discussion

This study introduced and developed an innovative, low-cost, modular smart headband integrated with textrodes for EEG measurement. Its performance was evaluated on spectral and ERP measures related to three standard tasks (alpha wave rhythms modulation for eyes-open vs eye-closed, P300 responses in an auditory oddball task, and P300 and N170 responses in a visual oddball task) and compared with a commercial sponge-based EEG cap. The smart headband was designed to enhance usability and comfort without compromising signal acquisition properties and latency. Findings demonstrate that the smart headband successfully captured the spectral and ERP specific signatures associated with each task. Moreover, these effects arise in very similar frequency/latency ranges in both the smart headband and the commercial EEG cap at the group level.

No post hoc or exploratory analyses were conducted; all reported validation targets presented in the paper (alpha band modulation for eyes-open/closed, auditory oddball P300, and visual face P300 and N170 detection) were the ones that we planned during the design of the experiment, based on previous studies [44]. This study deliberately limited the number of test protocols to three to ensure that all participants could perform all of them twice, additionally considering the time and potential discomfort of preparation and wearing both EEG systems one after the other. Therefore, all reported analyses were planned and executed as designed, without modification following inspection of the results.

Inter-device agreement was strongest for alpha power (ICC = 0.775, r = +0.782), consistent with the spatially broad and high-amplitude nature of alpha oscillations, which are inherently less sensitive to minor electrode placement differences than transient ERP components. Visual P300/N2 showed moderate individual-level agreement (ICC = 0.662, r = +0.744), while auditory P300 agreement was more limited (ICC = 0.508, r = +0.485). Visual N170 agreement was near zero (ICC = −0.167, r = −0.172), consistent with the known spatial sensitivity of this early component to electrode placement variability. Overall, the results support the feasibility of the smart headband integrated with textrodes has the potential for continuous ambulatory EEG measurement at the group level.

From a usability perspective, the smart headband was designed to enhance the ease of wearing and comfort while reducing device complexity through a minimal number of electrodes. The textrodes are soft and flexible, eliminating the need for electrolytic gel, which can cause skin irritation [13,14,70]. By eliminating the need for disposable gel electrodes and cumbersome wiring, the designed smart headband offers a reusable, adjustable, and more comfortable solution for long-term monitoring [12]. Survey findings related to comfort level suggested that this prototype design of a smart headband can be worn for long-term brain signal monitoring. Additionally, these textile-based textrodes reduce the costs associated with disposable gel-based medical-grade electrodes, are reusable, and can maintain hygienic compliance through multiple washes.

The current literature shows that a complex multi-channel EEG montage is used to maximize accuracy in laboratory and clinical studies [12]. From a usability perspective, wearers prefer EEG systems that are easy to wear, have minimal interfaces and wiring [44]. This study utilized a limited number of electrodes to perform the required measurements. However, when fewer electrode montages, the task-specific placement has a significant impact on the signals and the analysis of the behavioral tasks [44]. Nevertheless, encouraging results were obtained with this limited montage for alpha modulation, auditory, and visual stimulus paradigms.

***Limitations in the study:*** The fabrication of the textrode is straightforward, easy to adjust, cost-effective, and time efficient. Nevertheless, the pilot study presented comes with its limitations. First, limited ERP concordance was observed in this study. The smart headband and commercial cap were worn on separate occasions rather than simultaneously, introducing session-to-session variability in neural state and electrode contact. Furthermore, the headband places electrodes at positions that approximate but do not precisely replicate the temporal-parietal sites of the commercial cap. In addition, the small pilot sample further compromised the stability of individual-level agreement estimates (e.g., ICC estimation typically requires a minimum sample size of n = 30 [66]). Therefore, a small number of participants (n = 10) limits the generalizability and statistical power of analyses.

Second, the use of the smart headband for non-critical monitoring and screening applications could be validated at this stage, but could restrict its suitability for clinical diagnostics. Third, due to the intensive nature of laboratory experimentation, this study included only closed-ended questions related to comfort. Therefore, future research incorporating open-ended questions about the experience of wearing a smart headband would provide an opportunity to gain deeper insights into participants’ views on the smart headband design and its potential improvements. Finally, from a hardware perspective, the current setup utilized a Neuroelectrics microcontroller; future iterations could employ custom-designed or low-cost boards to reduce the cost of hardware setup further.

## VI. Conclusion and future research

In this study, a textile-based textrode-integrated smart headband was designed, fabricated, and evaluated. The application of this smart headband is straightforward and does not require any skin preparation. The super-soft and flexible design of the textrode ensures comfort for long-term EEG biopotential recordings. Furthermore, the modular design of the textrodes allows for adjustment according to the 10–20 international electrode placements, and the electrodes can be interchanged or removed for washing the headband, thereby maintaining hygienic compliance.

The findings illustrate that the neurocognitive responses detected from the smart headband are equivalent to those from the commercial EEG setup. However, the limited number of textrodes in the integrated headband may restrict the amount of information obtained compared to the commercial EEG cap. Nevertheless, this initial pilot study serves as proof of concept for EEG measurement using fabric-based soft textrodes, a future requirement for continuous monitoring using e-Textiles.

Future research will explore (i) the integration of additional textrodes into the smart headband in a modular and interchangeable headband design method, enabling an enhanced level of EEG measurements through a larger dataset of EEG measurements, and (ii) incorporating multimodal input sensing may offer improved accuracy for applications related to neurological disorders, such as epilepsy, Parkinson’s disease, unconscious state, sleep disorders, and autism-related conditions. Moreover, further focus on the integration and utilization of textrodes, in order to obtain multiple physiological signal measurements, such as ECG and EMG, will be carried out, which will lead to the creation of a wearable smart hybrid garment offering an unobtrusive wearable solution for health-related applications. This will expand the modular smart headband into a smart garment platform integrated with a BCI acquisition board that supports real-time electrode impedance measurement and further enhance signal quality assessment.
